# Similarities between the target and the intruder in naturally occurring repeated person naming errors

**DOI:** 10.3389/fpsyg.2015.01474

**Published:** 2015-09-29

**Authors:** Serge Brédart, Benoit Dardenne

**Affiliations:** ^1^Cognitive Psychology Unit, Department of Psychology, University of LiègeLiège, Belgium; ^2^Social Psychology Unit, Department of Psychology, University of LiègeLiège, Belgium

**Keywords:** person naming, phonological similarity, proper names, face resemblance, aging

## Abstract

The present study investigated an intriguing phenomenon that did not receive much attention so far: repeatedly calling a familiar person with someone else’s name. From participants’ responses to a questionnaire, these repeated naming errors were characterized with respect to a number of properties (e.g., type of names being substituted, error frequency, error longevity) and different features of similarity (e.g., age, gender, type of relationship with the participant, face resemblance and similarity of the contexts of encounter) between the bearer of the target name and the bearer of the wrong name. Moreover, it was evaluated whether the phonological similarity between names, the participants’ age, the difference of age between the two persons whose names were substituted, and face resemblance between the two persons predicted the frequency of error. Regression analyses indicated that phonological similarity between the target name and the wrong name predicted the frequency of repeated person naming errors. The age of the participant was also a significant predictor of error frequency: the older the participant the higher the frequency of errors. Consistent with previous research stressing the importance of the age of acquisition of words on lexical access in speech production, results indicated that bearer of the wrong name was on average known for longer than the bearer of the target name.

## Introduction

The failure to recall someone’s name at the right moment may be embarrassing. This everyday life difficulty can be uncomfortable for both the person who is unable to retrieve the name and the person whose name cannot be recalled. A large amount of research has demonstrated that names of persons are particularly likely to provoke retrieval difficulties (for a review see Griffin, 2010).

So far, the difficulty of person naming has been studied mainly through the analysis of naming failures such as tip-of-the-tongue states, and naming latencies (Hanley, 2014; Brédart, in press). Person naming errors received far less attention. Yet, calling someone by the wrong name is certainly as embarrassing as remaining unable to name a person at all, especially if the error is repeated over time. Repeated person naming errors are intriguing. Why do they perpetuate? A plausible hypothesis is that such errors go on because the target person and the holder of the intruding name share many important characteristics. Research on speech errors and more specifically on person naming errors revealed different kinds of similarities that are prone to elicit occasional naming errors.

Psycholinguistic studies of speech errors indicated that both phonological similarity and semantic similarity are factors favoring word substitutions. In addition, the combination of the two kinds of similarity (the *mixed semantic and phonological similarity effect*) was particularly prone to elicit word substitution (e.g., Dell and Reich, 1981; Martin et al., 1989, 1996). The significant effects of semantic similarity (sharing the same occupation) and phonological similarity, and the supra-additive effect of mixed semantic and phonological similarity were also reported in person naming (Brédart and Valentine, 1992). Moreover, a socio-cognitive study of person naming errors indicated that different similarities between the target person and the bearer of the wrong name on such factors as gender, age, and the category of relationship with the participant (e.g., friends, children, colleagues) increased the occurrence of name substitutions (Fiske et al., 1991). More recently, Griffin and Wangerman (2013) analyzed the characteristics of parents’ accidental speech errors consisting in calling a child with the name of a sibling. They showed that phonological similarity (same initial phoneme or same final phoneme) between the siblings’ names increased the frequency of name substitutions. In addition, high similarity of siblings’ physical appearance, same gender and similar age each contributed to increase the frequency of errors.

Most of previous studies of person misnaming have examined experimentally induced errors (Brédart and Valentine, 1992; Fraas et al., 2002) or occasional naturally occurring errors collected by questionnaire (Fiske et al., 1991; Griffin and Wangerman, 2013). The first aim of the present study was to describe the similarities accompanying the occurrence of repeated person naming errors on factors that have been found to increase occasional naming errors in previous studies, i.e., phonological similarity between the target name and the wrong name (Fiske et al., 1991; Brédart and Valentine, 1992; Fraas et al., 2002; Griffin and Wangerman, 2013), similarity of the relationship with the participant (Fiske et al., 1991), physical resemblance (Griffin and Wangerman, 2013) gender (Fiske et al., 1991; Griffin and Wangerman, 2013), and age similarity (Fiske et al., 1991; Griffin and Wangerman, 2013). The notion of physical resemblance is a little vague. Indeed, it may refer both to face resemblance and to body/silhouette resemblance. Because the face is probably the most distinctive physical feature of persons (McNeill, 1998; Tsakiris, 2008) and given that persons are more easily identified from their face than from their body (Burton et al., 1999; Bank et al., 2015), the role of face resemblance rather than physical similarity was studied here. A further factor that, intuitively, might participate in perpetuating errors was also examined: the similarity of the contexts of encounter. This study was also aimed at evaluating, when possible, which factors predicted the rated frequency of repeated errors.

In addition, given the vast literature demonstrating that a main determinant of lexical access in speech production is the age of acquisition of words (for a recent review see Brysbaert and Ellis, in press; for the specific case of face naming, see Moore and Valentine, 1998; Bonin et al., 2008), it was expected that intruding names would be acquired earlier than target names, and more specifically that holders of intruding names would be known for longer than holders of the target names.

Finally, an important result of previous research on person naming is that both the retrieval of known personal names and the learning of new names are impaired by normal aging (Burke et al., 1991; James, 2004, 2006; Old and Naveh-Benjamin, 2012). With respect to person naming errors, it has been reported that experimentally induced name substitutions occurred more frequently in older than in younger participants (Fraas et al., 2002). Hence, in the present study, it was evaluated whether or not aging is associated with an increase of the frequency of repeated naming errors.

In order to characterize what the main similarities associated with repeated naming errors are, participants who reported making repeated confusions between names were asked to choose one representative example and to characterize it in completing a questionnaire. Besides data on similarities, descriptive information was gathered about this undescribed kind of errors. Such descriptive information included the longevity of the error, the occurrence of a reverse error, the level of error monitoring, the frequency of encounter of the target and wrong persons, and the emotional valence attributed to the relationship with the target person and the intruder, respectively.

## Materials and Methods

### Participants

One hundred and eighty-one (110 females and 71 males) members of the administrative and technical staffs, *post doc* researchers, professors, senior researchers, and students from different departments of the University of Liège and the University Hospital, aged between 25 and 70 (*M* = 41.7; *SD* = 10.1), were screened with the following yes/no question: “Do you regularly confuse a particular person’s name with another person’s name?” 76 (46 females and 30 males) of them answered positively. All of them were recruited for the study. It is interesting to note that the mean age of participants who responded positively (*M* = 42.1; *SD* = 9.4) was not significantly different from the mean age of those who responded negatively (*M* = 41.6; *SD* = 10.6), Student *t-*test(179) = 0.42, *p* = 0.67 (two-tailed test). The average educational level measured by the number of years of study completed to achieve their highest degree was also similar in both groups (Yes: *M* = 18.5, *SD* = 3.3; No: *M* = 18.1, *SD* = 3.0), *t*-test(179) = 0.86, *p* = 0.39 (two-tailed test). This study was approved by the Ethics Committee of the Faculty of Psychology and Education of the University of Liège. All participants gave their written informed consent prior to participation.

### Procedure and Materials

The first author screened all the persons he met in the corridors of the University who accepted to be screened and belonged to the defined age range. People who responded positively to the screening question filled out a questionnaire under the supervision of the examiner within 2 weeks after the initial enquiry. Participants were instructed to focus on one representative case of repeated person naming error they used to make. The questionnaire invited the participants to provide the following pieces of information.

(1)  They were asked to write down both the target person’s name (A hereafter) and the wrong name (B) coming to mind.
(2)  They estimated the relative frequency of the four following ways in which the confusion may occur by assigning a percentage to each option so that the sum reaches 100%:

        –“You make an error by calling the target person by the wrong name and you do not spontaneously notice it” (uncorrected errors);
        –“You make an error by calling the target person by the wrong name but you notice the error and you self-repair” (corrected errors);
        –“You notice that you are going to make an error before making it and you produce the right name” (successful monitoring with correct naming);
        –“You notice that you are going to make an error before making it but the right name remains unavailable and you bypass the problem” (successful monitoring with naming failure).
(3)  They estimated (in years or in months) how long the error had been taking place.
(4)  They estimated the frequency of the error (each time they encounter the target person/more than half of the times/more than one out of five times/less frequently).
(5)  They provided information about their relation with A’s bearer: how long they had known the target person; how frequently they encountered that person (everyday/at least once a week/at least once a month/less frequently); the type of relationship with the person (professional/family/friendship/media person/other); the usual affective valence of the relation with that person on a 5-point scale (with -2 = negative, 0 = neutral, and 2 = positive).
(6)  They provided the same information as in five with respect to their relation with the B’ bearer.
(7)  They indicated whether they made also the reverse error, i.e., saying A instead of B on a 4-point scale with 1 = Never, 2 = Yes but less frequently, 3 = Yes as often, 4 = Yes, even more often.
(8)  They estimated the similarity between A’s bearer and B’s bearer with respect to facial similarity on 4-point scales with 1 = Not at all, 2 = Slight, 3 = Marked, 4 = Very strong. In addition, they estimated the similarity of the contexts in which they encounter the two persons, with 1 = Very different, 2 = Slightly different, 3 = Rather similar, 4 = Identical.
(9)  They indicated the age and gender of both A’s bearer and B’s bearer.

After the completion of the questionnaire, the participants were thanked and debriefed.

## Results

Among the 76 participants, 72 reported making naming errors while the other four reported confusion without naming error (successful error monitoring with correct naming). Substituted names were first names in 86.1% (62/72) of cases, full names in 8.3% (6/72), and surnames in the remaining 5.6% (4/72).

The average longevity of the errors was 5.5 years [95% CI 4.0, 6.9] (*SD* = 6.3) and most of them (76.3%; 58 out of 72 cases) still occurred currently. The reverse error (saying A for B) was judged as frequent as saying B for A in 54.2% (39/72) of cases, in 19.4% (14/72) of cases saying A for B was judged less frequent than saying B for A, in 1.4% (1/72) it was judged more frequent than saying B for A, in the remaining 25.0% (18/72) of cases the reverse error did not occur. There was no significant difference between the affective valence attributed to the relationship with A’s bearer and the affective valence attributed to the relationship with B’s bearer, Sign test, *z* = 1.66, *p* = 0.10.

A one-way ANOVA for repeated measures conducted on the percentages attributed to the four different categories of confusion revealed a significant difference, *F*(3,213) = 50.4; *p* < 0.0001. Planned comparisons (two-tailed tests) indicated that corrected errors [*M* = 56.5% (95% CI 50.0, 63.0), *SD* = 27.5] significantly outnumbered the other three categories [uncorrected errors, *M* = 17.8%, (95% CI 11.9, 23.7), *SD* = 25.0, *t*(71) = 7.1, *r* = 0.64; successful error monitoring with correct naming, *M* = 18.0% (95% CI 13.4, 22.6), *SD* = 19.6, *t*(71) = 8.3, *r* = 0.70; successful error monitoring with naming failure, *M* = 7.8% (95% CI 4.3, 11.2), *SD* = 14.8, *t*(71) = 11.4, *r* = 0.80], all *p*s < 0.001. There was no significant difference between uncorrected errors and successful error monitoring with correct naming, and these two categories were more frequent than successful error monitoring with naming failure, respectively, *t*(71) = 2.8, *r* = 0.31 and *t*(71) = 3.5, *r* = 0.39, *p*s < 0.01.

### Characterizing the Similarities that Accompanied Reported Repeated Naming Errors

The correspondence between the genders of the two persons was higher than expected by chance, in 68 out 72 cases the two persons had the same gender, Cohen’s Kappa = 0.89 [95% CI 0.78, 0.99], *z* = 1.94, *p* = 0.026. In three cases the participants called their son with their daughter’s name or vice versa and in the remaining case the participant called her sister with her boyfriend’s name.

The two persons whose names were substituted shared the same kind of relationship (categories: Professional relations; Family; Friendship; Neighbourship relations; Celebrities) with the participant more often than expected by chance (70 out of 72 cases), 0.95 (95% CI 0.89, 1.0), *z* = 3.39, *p* < 0.001. Substitutions between names of members of the participant’s family were numerically the most frequent (*n* = 41), followed by substitutions between names of persons encountered at work (*n* = 17), substitutions between friends’ names (*n* = 8), substitutions between famous people’ names (*n* = 3) and one substitution between neighbors’ names. The only two errors between the names of persons that did not share the same general relationship category were between a participant’s colleague and a childhood friend, and between a participant’s boss and her boyfriend.

The fact that, on the one hand, the mean age of A’s bearers (*M* = 24.6, *SD* = 16.0) and the mean age of B’s bearers (*M* = 26.1, *SD* = 16.6) were numerically similar and not significantly different, *t-*test(71) = 1.12, *p* = 0.27 (two-tailed test), and that, on the other hand, ages of A’s bearers and ages of B’s bearers correlated significantly, Pearson *r* = 0.76, *p* < 0.001, indicates that name substitutions mainly occurred for persons of similar ages.

On average, the index of phonological similarity (the number of phonemes shared by A and B divided by the number of phonemes in B) was 0.41 (95% CI 0.36, 0.47), *SD* = 0.24.

The level of facial similarity between A’s bearer and B’s bearer was globally weak. In more than three quarters of cases there was only a slight face resemblance (30.5%) or no face resemblance at all (47.2%) between the two persons whose names were substituted. There was a marked or a strong resemblance in 18.1% and 4.2% of cases, respectively. A chi square analysis indicated that this distribution was not random, χ^2^(3) = 29.0, *p* < 0.001. Complementary analyses indicated that the “no face resemblance at all” category was significantly more frequent than the categories “marked resemblance” and “strong resemblance”, respectively, χ^2^(1) = 9.4, *p* = 0.002 and χ^2^(1) = 25.9, *p* < 0.001. In addition, the category “strong resemblance” was significantly less frequent than the “slight face resemblance” and “marked face resemblance” categories, respectively, χ^2^(1) = 14.4, *p* < 0.001 and χ^2^(1) = 6.3, *p* = 0.012. There was no other significant difference.

By contrast the level of similarity of the contexts in which A’s bearer and B’s bearer were encountered was globally high. In more than 90% of reported errors, A’s bearer and B’s bearers were usually encountered in either rather similar (26.4%) or identical contexts (63.9%). The two persons were encountered in slightly different or very different contexts in 4.2% and 5.7%, respectively. A chi square analysis indicated that this distribution was not random, χ^2^(3) = 67.6, *p* < 0.001. Complementary analyses indicated that the “identical contexts” category was significantly more frequent than the categories “rather similar contexts”, χ^2^(1) = 11.2, *p* < 0.001, “slightly different contexts”, χ^2^(1) = 35.3, *p* < 0.001, and “very different contexts” χ^2^(1) = 25.9, *p* < 0.001. Moreover, the category “rather similar contexts” was significantly more frequent than the “slightly different contexts” and “very different contexts”, respectively, χ^2^(1) = 11.6, *p* < 0.001 and χ^2^(1) = 9.8, *p* = 0.002. No other difference was significant.

### Which Similarities Predict the Frequency of Errors?

The role of the following factors was tested: the phonological similarity between the names (the predictor was the number of phonemes shared by the two names divided by the number of phonemes in B), face resemblance, the difference of age (the predictor was the absolute difference between the age of A and the age of B), and the age of the participant. Unfortunately it was not possible to include the gender as a predictor. Indeed, since in 94.4% of cases, errors occurred between names of same gender persons, this factor was almost constant. The lack of variance also precluded the inclusion of the similarity of relationship with the participant as a predictor: in 97.2% of cases errors occurred between names of persons sharing the same type of relationship with the participant. For the same reason, context similarity was not included as a predictor (the similarity was high in more than 90% of cases).

In order to eliminate categories with too few observations, responses *each time they encounter the target person* (9.7%) and *more than half of the times* (18.1%) for the estimation of error frequency were collapsed into one single category labeled *often*. So, analyses were conducted on whether participants reported making the error *often* (coded as 3; 28% of responses), *more than one out of five times* (coded as 2; 29% of responses), *less frequently* (coded as 1; 43% of responses). The ratings of face resemblance were also reduced to three levels (instead of four): ratings *very strong* were merged with *marked* (for a total of 22.2% of responses). Face resemblance was treated as an ordinal predictor, while the age of the participants, age difference between A’s and B’s bearers, and phonological similarity were treated as continuous variables. All continuous predictors except the interaction between age of the participants and phonological similarity were then mean-centered.

Data were analyzed with ordered (cumulative) logit regression for ordinal responses (see Agresti, 2007). This analysis estimated the impact of a predictor on the odds of being at or above an outcome variable category (for instance, at *more than one out of five times* or *often*). Brant test of parallel lines provided evidence that the location parameters (slope coefficients) were the same across the three response categories, χ^2^(6) = 3.85, *p* = 0.69. **Table 1** displays the parameters for the model.

**Table 1:** Parameters for the estimated cumulative ordinal regression model for error frequency.

Predictor	*b (log-odds)*	*SE(b)*	Odds ratio	Wald χ^2^	*df*	*p*
Face similarity (1 vs. 3)	-0.411	0.341	0.663	1.45	1	0.228
Face similarity (2 vs. 3)	0.808	0.385	2.443	4.40	1	0.036
Age of participants	0.050	0.026	1.051	3.62	1	0.028^∗^
Age difference A-B	-0.048	0.030	0.953	2.62	1	0.106
Phonological similarity	2.851	1.147	17.305	6.17	1	0.013
Age Pp × Phonological similarity	-0.073	0.111	0.930	0.43	1	0.513
Intercept (*infrequently* vs. *more than one out of five times* and *often*)	-0.257	0.279				
Intercept (*infrequently* and *more than one out of five times* vs. *often*)	1.309	0.320				

*N = 72. Model fitting information -2 Log Likelihood χ^2^(6) = 21.52, p < 0.001. Nagelkerke pseudo R^2^ = 0.292. ^∗^One-tailed test*.

Although the overall Wald statistic for face resemblance did not come up significant, χ^2^(2) = 4.59, *p* = 0.10, there was a significant increase in error frequency when going from *slight* resemblance (category 2) to the highest facial resemblance (category 3). The odds of reporting an increase in error frequency were 2.44 times greater when A and B bearers shared a high facial similarity compared to just a slight facial similarity (see **Table 1**). Phonological similarity appeared to be a strong predictor of error frequency. A one unit increase in phonological similarity was associated with a 2.85 increase in log-odds of being in a higher error frequency category with other variables in the model held constant. Stated in another words, the odds of reporting an increase in error frequency was 17 times greater for a one unit increase in phonological similarity. For each additional unit increase in phonological similarity, there was a 94.5% chance to get into the higher frequency of error category. The difference between the age of A’s bearer and B’s bearer failed to reach statistical significance (*p* = 0.106).

### Was B’s Bearer Known for Longer than A’s Bearer?

A Student *t*-test for paired samples indicated that B’s bearers were significantly known for longer [*M* = 12.8 years (95% CI 9.8, 15.9), *SD* = 12.9] than A’s bearers [*M* = 9.9 years (95% CI 7.7, 12.1), *SD* = 9.2], *t*(71) = 2.45, *p* = 0.017, *r* = 0.30. However, the frequency of encounter did not differ between A bearers (*M* = 1.9, *SD* = 1.1) and B bearers (*M* = 2.0, *SD* = 1.1), Sign test, *z* = 1.07, *p* = 0.28 (1 = everyday; 2 = at least once a week; 3 = at least once a month; 4 = less frequently).

### Was the Age of the Participants Associated with an Increase of the Frequency of Repeated Naming Errors?

The logit regression mentioned above also included the age of the participants as a predictor. The analysis indicated that ten additional years of participants’ age were associated with about a 12.5% increase in error frequency (1.25% for each year of age of a participant; OR = 1.051; *p* = 0.028 one-tailed test) but there was no evidence of an Age × Phonological similarity interaction.

## Discussion

The present study was aimed at characterizing naturally occurring repeated person naming errors, a phenomenon that has not been studied much. The errors reported by our participants were in the vast majority substitutions between first names. The average longevity of these errors was 5 years and an half, most of these errors currently continuing. The bearers of the target and of the intruding names were same-sex persons and shared the same type of general relationship (e.g., professional, family, friendship, or neighbourship relation) with the participant more frequently than expected by chance. They were of similar ages, and were most often encountered in similar or identical contexts. By contrast, there was mainly a weak similarity or no similarity at all between the target person and the intruder with respect to facial appearance.

As expected from previous literature on the occurrence of experimentally induced occasional naming errors (Dell and Reich, 1981; Martin et al., 1989, 1996; Brédart and Valentine, 1992), phonological similarity was a significant predictor of the frequency of errors: the higher the number of phonemes shared by the target name and the intruding name, the higher the frequency of errors. Another expected predictor was semantic relatedness. It was planned to evaluate whether the fact that A’s bearer and B’s bearer shared the same type of relationship with the participant impacted the frequency of errors. However, the data indicated that the virtually all errors occurred while A’s bearer and B’s bearer had the same type of general relationship with the participant. Consequently, it was not possible to assess the role of semantic relatedness. Another consequence of this lack of variability is that it was impossible to assess directly the mixed semantic and phonological similarity effect. Just like Griffin and Wangerman (2013), we nevertheless show that phonological similarity increased the frequency of errors between names of semantically related people. Our study did not allow comparing the impact of phonological similarity on errors between names of semantically related persons to that of phonological similarity on errors between names of semantically unrelated persons. Without such a comparison, it is not possible to determine what kind of interactive or discrete model of naming our data fit better (Rapp and Goldrick, 2000).

Although face resemblance was not globally a significant predictor of error frequency, there was a significant increase in error frequency from slight resemblance to the highest facial resemblance. In Griffin and Wangerman (2013), physical similarity (a related but wider concept than facial resemblance, see also Introduction) was found to be a significant predictor of error frequency. The relative difference between the Griffin and Wangerman (2013) study and ours could stem from the fact that, in our study, the level of face resemblance was low for more than three quarters of errors. Given that substituted names were siblings’ names in Griffin and Wangerman (2013), the overall level of face resemblance was presumably higher in that study than in the present one, and the distribution of errors across the different levels of resemblance was better balanced than in our study.

At odds with Griffin and Wangerman’s (2013) study, the difference of age between the bearer of the target and the bearer of the wrong name was not a significant predictor. This discrepancy could be explained by a major difference between the two studies with respect to age determination. People usually know the true age of their siblings but they often estimate the age of less well known people such as colleagues or actors. We checked the true ages of celebrities involved in the reported errors. Here are two striking examples. Isabelle Adjani’s age was estimated at 54 years and Sophie Marceau age’s at 50. True ages of these actresses are, respectively, 60 and 49. The true age difference was 11 and not 4 as estimated by the participant. Charles Michel’s (current Prime Minister of Belgium) age was estimated at 45 years, his true age is 39. Louis Michel’s (current member of the European Parliament and State Minister of Belgium) age was estimated at 64 and is really 67; in this case the true age difference was 28 years and not 19. We also found cases where the age difference was overestimated. The lack of age estimation accuracy might have blurred the relation between the real age difference and error frequency.

Based on studies that demonstrated the role of the age-of-acquisition of names in person naming (Moore and Valentine, 1998; Bonin et al., 2008), it was predicted that bearers of the intruding names would be known for longer than bearers of the target names. Results confirmed that prediction.

Finally, Fraas et al. (2002) reported that experimentally induced name substitutions occurred more frequently in older than in younger participants (see also Griffin and Wangerman, 2013, p. 4). The present study extends this result in showing that the frequency of naturally occurring repeated naming errors increased with age.

In sum, similarly to previous studies on occasional naming errors (Fiske et al., 1991; Brédart and Valentine, 1992; Fraas et al., 2002; Griffin and Wangerman, 2013), the present study showed that the age of the participant and phonological similarity between the target name and the intruding name were significant predictors of repeated person naming error frequency. In future studies, a direct comparison of repeated and occasional person naming errors by using a questionnaire addressing both kinds of errors should help understanding further the specificities of repeated errors.

## Conflict of Interest Statement

The authors declare that the research was conducted in the absence of any commercial or financial relationships that could be construed as a potential conflict of interest.
